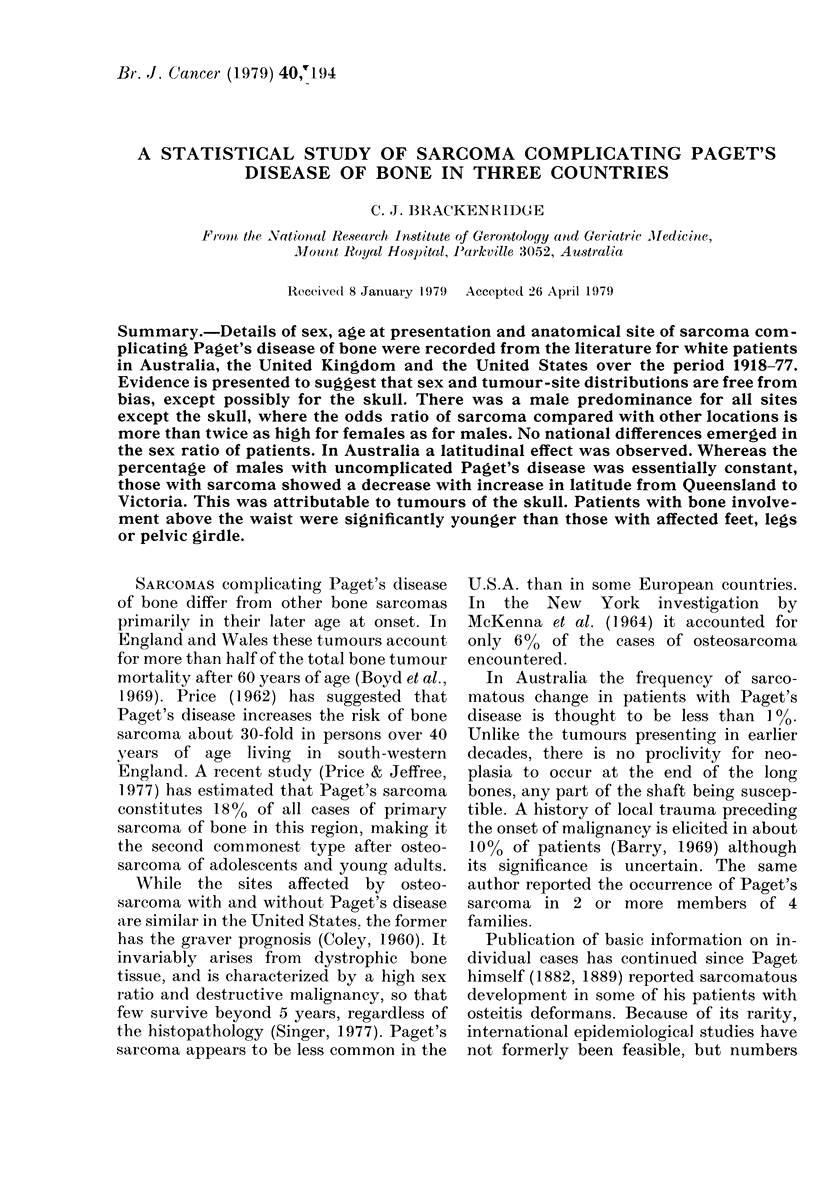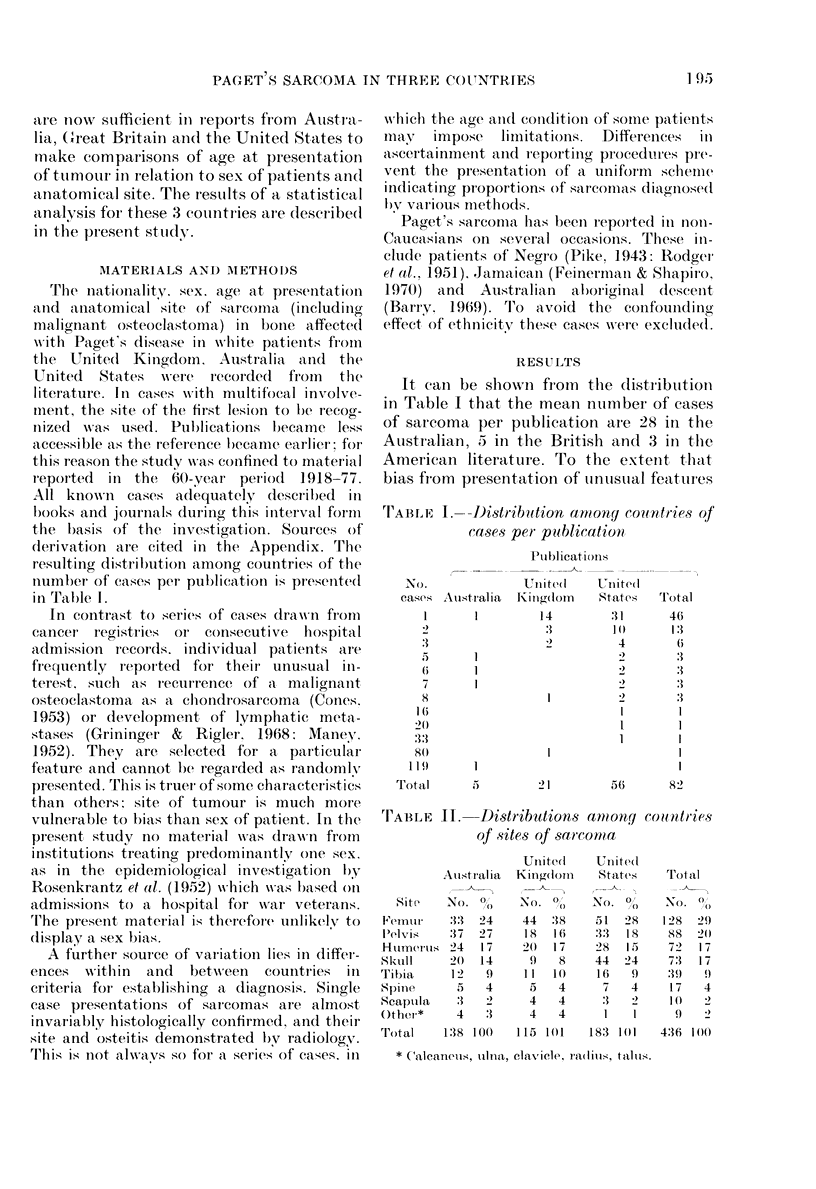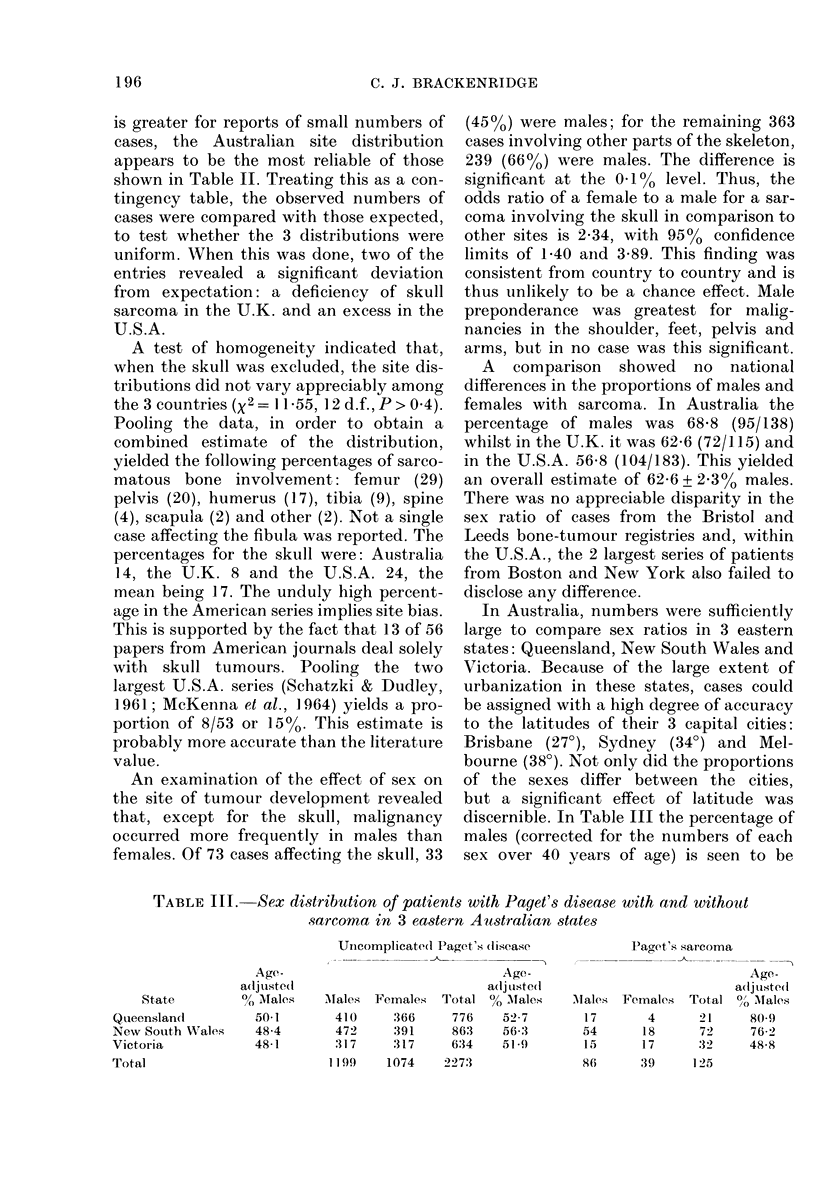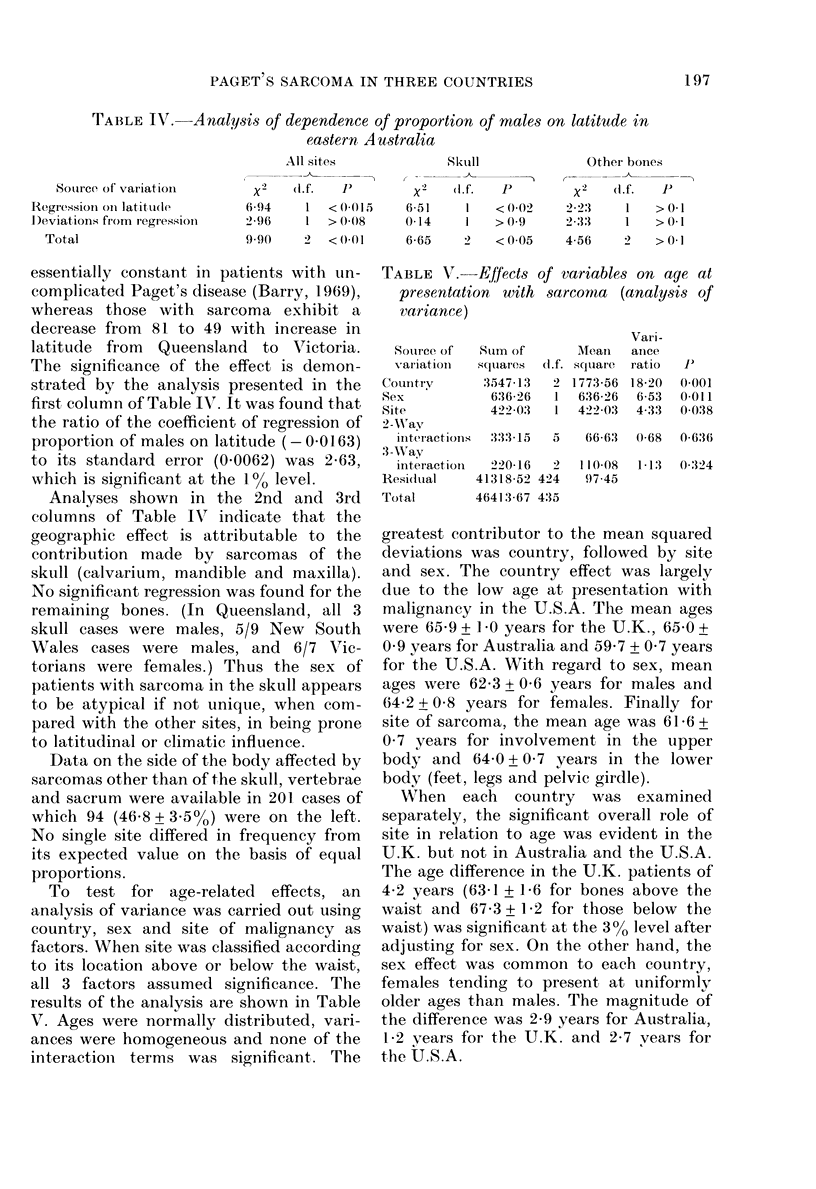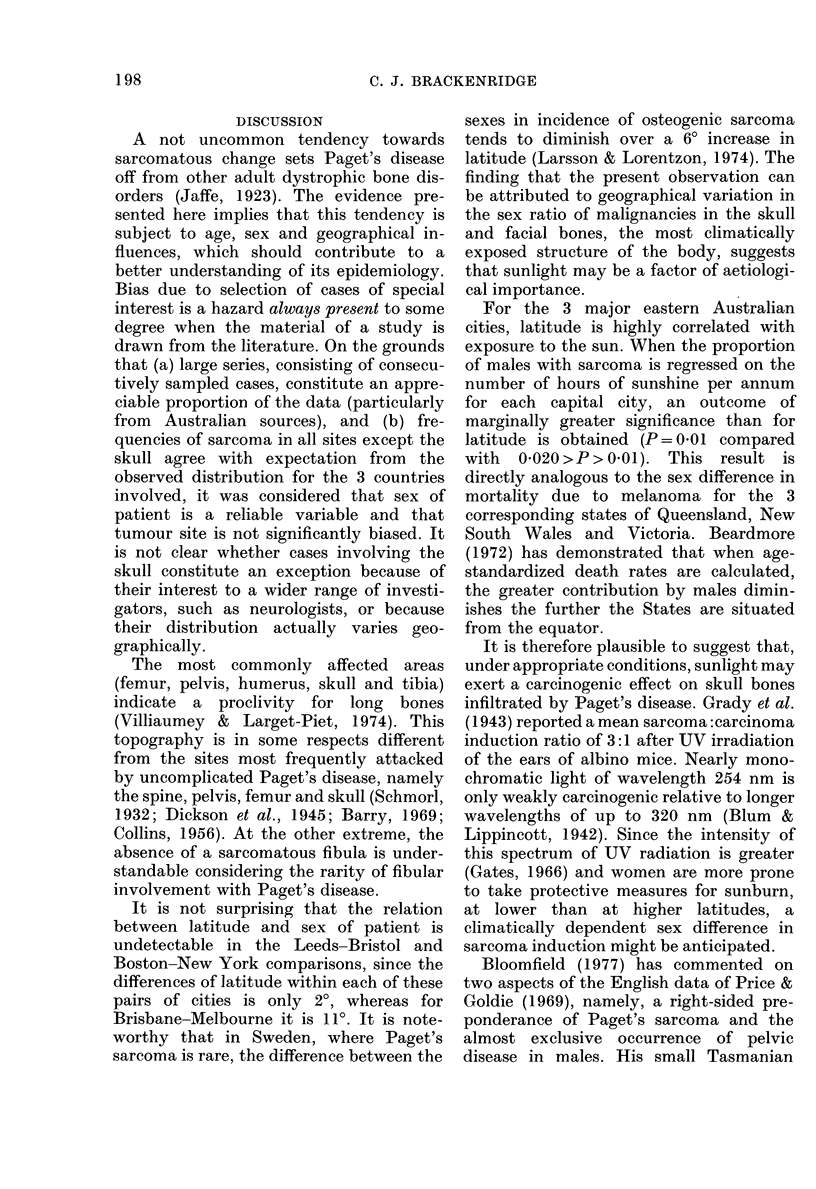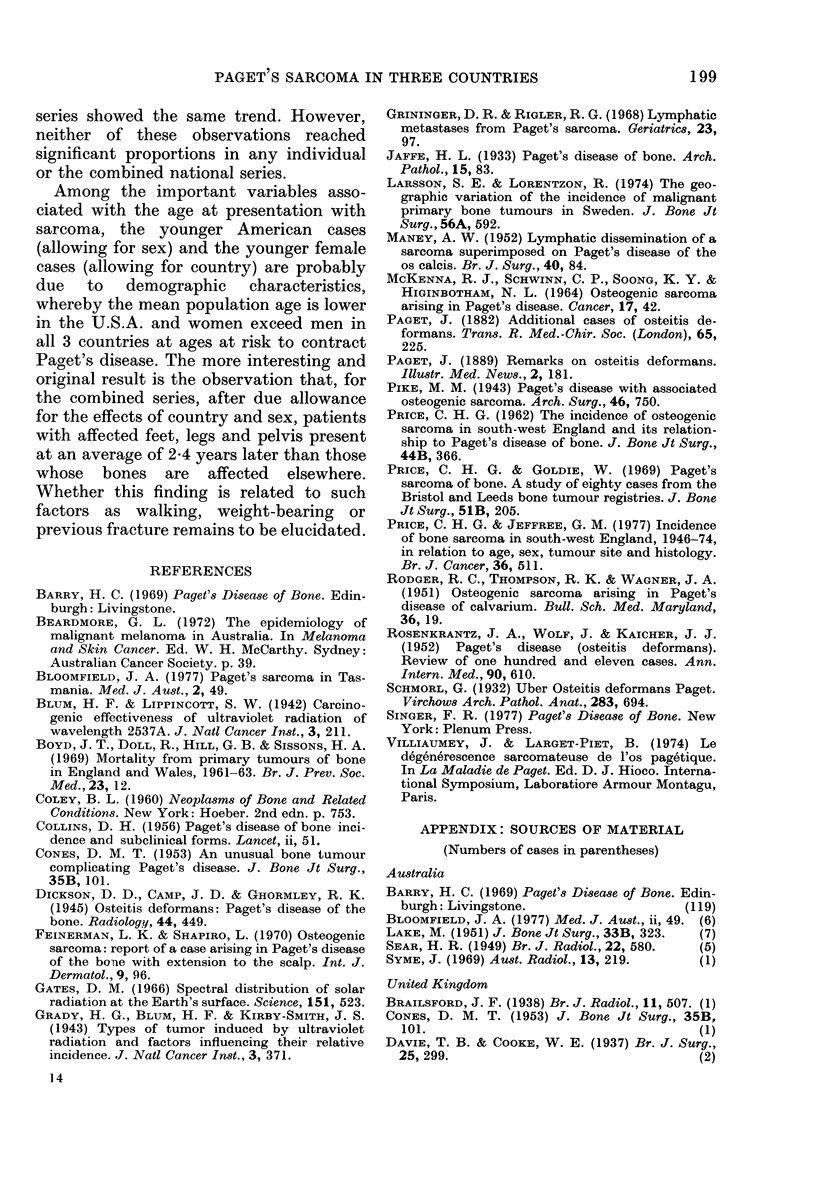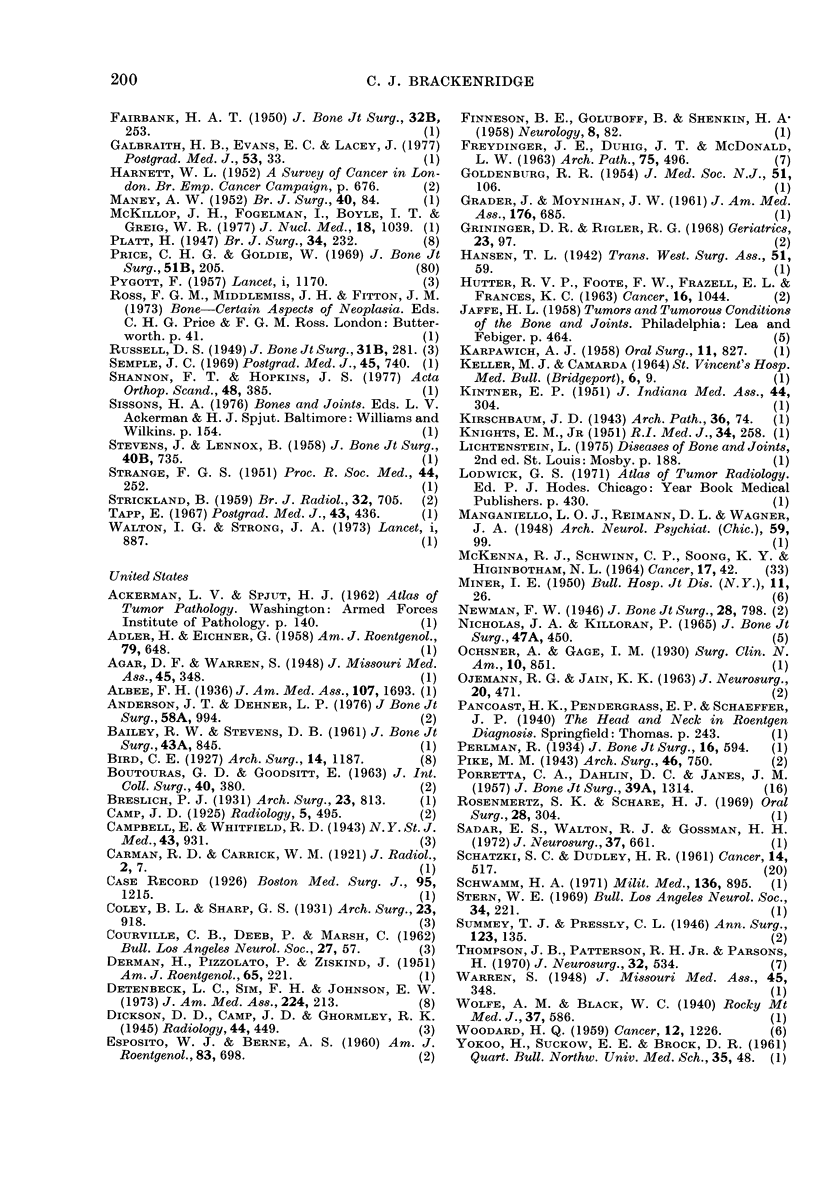# A statistical study of sarcoma complicating Paget's disease of bone in three countries.

**DOI:** 10.1038/bjc.1979.166

**Published:** 1979-08

**Authors:** C. J. Brackenridge

## Abstract

Details of sex, age at presentation and anatomical site of sarcoma complicating Paget's disease of bone were recorded from the literature for white patients in Australia, the United Kingdom and the United States over the period 1978--77. Evidence is presented to suggest that sex and tumour-site distributions are free from bias, except possibly for the skull. There was a male predominance for all sites except the skull, where the odds ratio of sarcoma compared with other locations is more than twice as high for females as for males. No national differences emerged in the sex ratio of patients. In Australia a latitudinal effect was observed. Whereas the percentage of males with uncomplicated Paget's disease was essentially constant, those with sarcoma showed a decrease with increase in latitude from Queensland to Victoria. This was attributable to tumours of the skull. Patients with bone involvement above the waist were significantly younger than those with affected feet, legs or pelvic girdle.


*Br. J. Cancer* (1979) **40**, 194

### A STATISTICAL STUDY OF SARCOMA COMPLICATING PAGET'S DISEASE OF BONE IN THREE COUNTRIES

C. J. BRACKENRIDGE

*From the National Research Institute of Gerontology and Geriatric Medicine, Mount Royal Hospital, Parkville 3052, Australia*

Received 8 January 1979 Accepted 26 April 1979

**Summary.—Details of sex, age at presentation and anatomical site of sarcoma complicating Paget's disease of bone were recorded from the literature for white patients in Australia, the United Kingdom and the United States over the period 1918–77. Evidence is presented to suggest that sex and tumour-site distributions are free from bias, except possibly for the skull. There was a male predominance for all sites except the skull, where the odds ratio of sarcoma compared with other locations is more than twice as high for females as for males. No national differences emerged in the sex ratio of patients. In Australia a latitudinal effect was observed. Whereas the percentage of males with uncomplicated Paget's disease was essentially constant, those with sarcoma showed a decrease with increase in latitude from Queensland to Victoria. This was attributable to tumours of the skull. Patients with bone involvement above the waist were significantly younger than those with affected feet, legs or pelvic girdle.**

SARCOMAS complicating Paget's disease of bone differ from other bone sarcomas primarily in their later age at onset. In England and Wales these tumours account for more than half of the total bone tumour mortality after 60 years of age (Boyd *et al.*, 1969). Price (1962) has suggested that Paget's disease increases the risk of bone sarcoma about 30-fold in persons over 40 years of age living in south-western England. A recent study (Price & Jeffree, 1977) has estimated that Paget's sarcoma constitutes 18% of all cases of primary sarcoma of bone in this region, making it the second commonest type after osteosarcoma of adolescents and young adults.

While the sites affected by osteosarcoma with and without Paget's disease are similar in the United States, the former has the graver prognosis (Coley, 1960). It invariably arises from dystrophic bone tissue, and is characterized by a high sex ratio and destructive malignancy, so that few survive beyond 5 years, regardless of the histopathology (Singer, 1977). Paget's sarcoma appears to be less common in the U.S.A. than in some European countries. In the New York investigation by McKenna *et al.* (1964) it accounted for only 6% of the cases of osteosarcoma encountered.

In Australia the frequency of sarcomatous change in patients with Paget's disease is thought to be less than 1%. Unlike the tumours presenting in earlier decades, there is no proclivity for neoplasia to occur at the end of the long bones, any part of the shaft being susceptible. A history of local trauma preceding the onset of malignancy is elicited in about 10% of patients (Barry, 1969) although its significance is uncertain. The same author reported the occurrence of Paget's sarcoma in 2 or more members of 4 families.

Publication of basic information on individual cases has continued since Paget himself (1882, 1889) reported sarcomatous development in some of his patients with osteitis deformans. Because of its rarity, international epidemiological studies have not formerly been feasible, but numbers are now sufficient in reports from Australia, Great Britain and the United States to make comparisons of age at presentation of tumour in relation to sex of patients and anatomical site. The results of a statistical analysis for these 3 countries are described in the present study.

### MATERIALS AND METHODS

The nationality, sex, age at presentation and anatomical site of sarcoma (including malignant osteoclastoma) in bone affected with Paget's disease in white patients from the United Kingdom, Australia and the United States were recorded from the literature. In cases with multifocal involvement, the site of the first lesion to be recognized was used. Publications became less accessible as the reference became earlier; for this reason the study was confined to material reported in the 60-year period 1918–77. All known cases adequately described in books and journals during this interval form the basis of the investigation. Sources of derivation are cited in the Appendix. The resulting distribution among countries of the number of cases per publication is presented in Table I.

In contrast to series of cases drawn from cancer registries or consecutive hospital admission records, individual patients are frequently reported for their unusual interest, such as recurrence of a malignant osteoclastoma as a chondrosarcoma (Cones, 1953) or development of lymphatic metastases (Grininger & Rigler, 1968; Maney, 1952). They are selected for a particular feature and cannot be regarded as randomly presented. This is truer of some characteristics than others; site of tumour is much more vulnerable to bias than sex of patient. In the present study no material was drawn from institutions treating predominantly one sex, as in the epidemiological investigation by Rosenkrantz *et al.* (1952) which was based on admissions to a hospital for war veterans. The present material is therefore unlikely to display a sex bias.

A further source of variation lies in differences within and between countries in criteria for establishing a diagnosis. Single case presentations of sarcomas are almost invariably histologically confirmed, and their site and osteitis demonstrated by radiology. This is not always so for a series of cases, in which the age and condition of some patients may impose limitations. Differences in ascertainment and reporting procedures prevent the presentation of a uniform scheme indicating proportions of sarcomas diagnosed by various methods.

Paget's sarcoma has been reported in non-Caucasians on several occasions. These include patients of Negro (Pike, 1943; Rodger *et al.*, 1951), Jamaican (Feinerman & Shapiro, 1970) and Australian aboriginal descent (Barry, 1969). To avoid the confounding effect of ethnicity these cases were excluded.

### RESULTS

It can be shown from the distribution in Table I that the mean number of cases of sarcoma per publication are 28 in the Australian, 5 in the British and 3 in the American literature. To the extent that bias from presentation of unusual features

TABLE I.—*Distribution among countries of cases per publication*

| | Publications | | | |
|---|---|---|---|---|
| No. cases | Australia | United Kingdom | United States | Total |
| 1 | 1 | 14 | 31 | 46 |
| 2 | | 3 | 10 | 13 |
| 3 | | 2 | 4 | 6 |
| 5 | 1 | | 2 | 3 |
| 6 | 1 | | 2 | 3 |
| 7 | 1 | | 2 | 3 |
| 8 | | 1 | 2 | 3 |
| 16 | | | 1 | 1 |
| 20 | | | 1 | 1 |
| 33 | | | 1 | 1 |
| 80 | | 1 | | 1 |
| 119 | 1 | | | 1 |
| Total | 5 | 21 | 56 | 82 |

TABLE II.—*Distributions among countries of sites of sarcoma*

| | Australia | | United Kingdom | | United States | | Total | |
|---|---|---|---|---|---|---|---|---|
| Site | No. | % | No. | % | No. | % | No. | % |
| Femur | 33 | 24 | 44 | 38 | 51 | 28 | 128 | 29 |
| Pelvis | 37 | 27 | 18 | 16 | 33 | 18 | 88 | 20 |
| Humerus | 24 | 17 | 20 | 17 | 28 | 15 | 72 | 17 |
| Skull | 20 | 14 | 9 | 8 | 44 | 24 | 73 | 17 |
| Tibia | 12 | 9 | 11 | 10 | 16 | 9 | 39 | 9 |
| Spine | 5 | 4 | 5 | 4 | 7 | 4 | 17 | 4 |
| Scapula | 3 | 2 | 4 | 4 | 3 | 2 | 10 | 2 |
| Other* | 4 | 3 | 4 | 4 | 1 | 1 | 9 | 2 |
| Total | 138 | 100 | 115 | 101 | 183 | 101 | 436 | 100 |

* Calcaneus, ulna, clavicle, radius, talus.

is greater for reports of small numbers of cases, the Australian site distribution appears to be the most reliable of those shown in Table II. Treating this as a contingency table, the observed numbers of cases were compared with those expected, to test whether the 3 distributions were uniform. When this was done, two of the entries revealed a significant deviation from expectation: a deficiency of skull sarcoma in the U.K. and an excess in the U.S.A.

A test of homogeneity indicated that, when the skull was excluded, the site distributions did not vary appreciably among the 3 countries ($\chi^2 = 11{\cdot}55$, 12 d.f., $P > 0{\cdot}4$). Pooling the data, in order to obtain a combined estimate of the distribution, yielded the following percentages of sarcomatous bone involvement: femur (29) pelvis (20), humerus (17), tibia (9), spine (4), scapula (2) and other (2). Not a single case affecting the fibula was reported. The percentages for the skull were: Australia 14, the U.K. 8 and the U.S.A. 24, the mean being 17. The unduly high percentage in the American series implies site bias. This is supported by the fact that 13 of 56 papers from American journals deal solely with skull tumours. Pooling the two largest U.S.A. series (Schatzki & Dudley, 1961; McKenna *et al.*, 1964) yields a proportion of 8/53 or 15%. This estimate is probably more accurate than the literature value.

An examination of the effect of sex on the site of tumour development revealed that, except for the skull, malignancy occurred more frequently in males than females. Of 73 cases affecting the skull, 33 (45%) were males; for the remaining 363 cases involving other parts of the skeleton, 239 (66%) were males. The difference is significant at the 0·1% level. Thus, the odds ratio of a female to a male for a sarcoma involving the skull in comparison to other sites is 2·34, with 95% confidence limits of 1·40 and 3·89. This finding was consistent from country to country and is thus unlikely to be a chance effect. Male preponderance was greatest for malignancies in the shoulder, feet, pelvis and arms, but in no case was this significant.

A comparison showed no national differences in the proportions of males and females with sarcoma. In Australia the percentage of males was 68·8 (95/138) whilst in the U.K. it was 62·6 (72/115) and in the U.S.A. 56·8 (104/183). This yielded an overall estimate of $62{\cdot}6 \pm 2{\cdot}3\%$ males. There was no appreciable disparity in the sex ratio of cases from the Bristol and Leeds bone-tumour registries and, within the U.S.A., the 2 largest series of patients from Boston and New York also failed to disclose any difference.

In Australia, numbers were sufficiently large to compare sex ratios in 3 eastern states: Queensland, New South Wales and Victoria. Because of the large extent of urbanization in these states, cases could be assigned with a high degree of accuracy to the latitudes of their 3 capital cities: Brisbane (27°), Sydney (34°) and Melbourne (38°). Not only did the proportions of the sexes differ between the cities, but a significant effect of latitude was discernible. In Table III the percentage of males (corrected for the numbers of each sex over 40 years of age) is seen to be

TABLE III.—*Sex distribution of patients with Paget's disease with and without sarcoma in 3 eastern Australian states*

| | | Uncomplicated Paget's disease | | | | Paget's sarcoma | | | |
|---|---|---|---|---|---|---|---|---|---|
| State | Age-adjusted % Males | Males | Females | Total | Age-adjusted % Males | Males | Females | Total | Age-adjusted % Males |
| Queensland | 50·1 | 410 | 366 | 776 | 52·7 | 17 | 4 | 21 | 80·9 |
| New South Wales | 48·4 | 472 | 391 | 863 | 56·3 | 54 | 18 | 72 | 76·2 |
| Victoria | 48·1 | 317 | 317 | 634 | 51·9 | 15 | 17 | 32 | 48·8 |
| Total | | 1199 | 1074 | 2273 | | 86 | 39 | 125 | |

TABLE IV.—*Analysis of dependence of proportion of males on latitude in eastern Australia*

| Source of variation | All sites $\chi^2$ | d.f. | $P$ | Skull $\chi^2$ | d.f. | $P$ | Other bones $\chi^2$ | d.f. | $P$ |
|---|---|---|---|---|---|---|---|---|---|
| Regression on latitude | 6·94 | 1 | $< 0·015$ | 6·51 | 1 | $< 0·02$ | 2·23 | 1 | $> 0·1$ |
| Deviations from regression | 2·96 | 1 | $> 0·08$ | 0·14 | 1 | $> 0·9$ | 2·33 | 1 | $> 0·1$ |
| Total | 9·90 | 2 | $< 0·01$ | 6·65 | 2 | $< 0·05$ | 4·56 | 2 | $> 0·1$ |

essentially constant in patients with uncomplicated Paget's disease (Barry, 1969), whereas those with sarcoma exhibit a decrease from 81 to 49 with increase in latitude from Queensland to Victoria. The significance of the effect is demonstrated by the analysis presented in the first column of Table IV. It was found that the ratio of the coefficient of regression of proportion of males on latitude ($-0·0163$) to its standard error (0·0062) was 2·63, which is significant at the 1% level.

Analyses shown in the 2nd and 3rd columns of Table IV indicate that the geographic effect is attributable to the contribution made by sarcomas of the skull (calvarium, mandible and maxilla). No significant regression was found for the remaining bones. (In Queensland, all 3 skull cases were males, 5/9 New South Wales cases were males, and 6/7 Victorians were females.) Thus the sex of patients with sarcoma in the skull appears to be atypical if not unique, when compared with the other sites, in being prone to latitudinal or climatic influence.

Data on the side of the body affected by sarcomas other than of the skull, vertebrae and sacrum were available in 201 cases of which 94 ($46·8 \pm 3·5\%$) were on the left. No single site differed in frequency from its expected value on the basis of equal proportions.

To test for age-related effects, an analysis of variance was carried out using country, sex and site of malignancy as factors. When site was classified according to its location above or below the waist, all 3 factors assumed significance. The results of the analysis are shown in Table V. Ages were normally distributed, variances were homogeneous and none of the interaction terms was significant. The

TABLE V.—*Effects of variables on age at presentation with sarcoma (analysis of variance)*

| Source of variation | Sum of squares | d.f. | Mean square | Variance ratio | $P$ |
|---|---|---|---|---|---|
| Country | 3547·13 | 2 | 1773·56 | 18·20 | 0·001 |
| Sex | 636·26 | 1 | 636·26 | 6·53 | 0·011 |
| Site | 422·03 | 1 | 422·03 | 4·33 | 0·038 |
| 2-Way interactions | 333·15 | 5 | 66·63 | 0·68 | 0·636 |
| 3-Way interaction | 220·16 | 2 | 110·08 | 1·13 | 0·324 |
| Residual | 41318·52 | 424 | 97·45 | | |
| Total | 46413·67 | 435 | | | |

greatest contributor to the mean squared deviations was country, followed by site and sex. The country effect was largely due to the low age at presentation with malignancy in the U.S.A. The mean ages were $65·9 \pm 1·0$ years for the U.K., $65·0 \pm 0·9$ years for Australia and $59·7 \pm 0·7$ years for the U.S.A. With regard to sex, mean ages were $62·3 \pm 0·6$ years for males and $64·2 \pm 0·8$ years for females. Finally for site of sarcoma, the mean age was $61·6 \pm 0·7$ years for involvement in the upper body and $64·0 \pm 0·7$ years in the lower body (feet, legs and pelvic girdle).

When each country was examined separately, the significant overall role of site in relation to age was evident in the U.K. but not in Australia and the U.S.A. The age difference in the U.K. patients of 4·2 years ($63·1 \pm 1·6$ for bones above the waist and $67·3 \pm 1·2$ for those below the waist) was significant at the 3% level after adjusting for sex. On the other hand, the sex effect was common to each country, females tending to present at uniformly older ages than males. The magnitude of the difference was 2·9 years for Australia, 1·2 years for the U.K. and 2·7 years for the U.S.A.

### DISCUSSION

A not uncommon tendency towards sarcomatous change sets Paget's disease off from other adult dystrophic bone disorders (Jaffe, 1923). The evidence presented here implies that this tendency is subject to age, sex and geographical influences, which should contribute to a better understanding of its epidemiology. Bias due to selection of cases of special interest is a hazard *always present* to some degree when the material of a study is drawn from the literature. On the grounds that (a) large series, consisting of consecutively sampled cases, constitute an appreciable proportion of the data (particularly from Australian sources), and (b) frequencies of sarcoma in all sites except the skull agree with expectation from the observed distribution for the 3 countries involved, it was considered that sex of patient is a reliable variable and that tumour site is not significantly biased. It is not clear whether cases involving the skull constitute an exception because of their interest to a wider range of investigators, such as neurologists, or because their distribution actually varies geographically.

The most commonly affected areas (femur, pelvis, humerus, skull and tibia) indicate a proclivity for long bones (Villiaumey & Larget-Piet, 1974). This topography is in some respects different from the sites most frequently attacked by uncomplicated Paget's disease, namely the spine, pelvis, femur and skull (Schmorl, 1932; Dickson *et al.*, 1945; Barry, 1969; Collins, 1956). At the other extreme, the absence of a sarcomatous fibula is understandable considering the rarity of fibular involvement with Paget's disease.

It is not surprising that the relation between latitude and sex of patient is undetectable in the Leeds–Bristol and Boston–New York comparisons, since the differences of latitude within each of these pairs of cities is only 2°, whereas for Brisbane–Melbourne it is 11°. It is noteworthy that in Sweden, where Paget's sarcoma is rare, the difference between the sexes in incidence of osteogenic sarcoma tends to diminish over a 6° increase in latitude (Larsson & Lorentzon, 1974). The finding that the present observation can be attributed to geographical variation in the sex ratio of malignancies in the skull and facial bones, the most climatically exposed structure of the body, suggests that sunlight may be a factor of aetiological importance.

For the 3 major eastern Australian cities, latitude is highly correlated with exposure to the sun. When the proportion of males with sarcoma is regressed on the number of hours of sunshine per annum for each capital city, an outcome of marginally greater significance than for latitude is obtained ($P = 0{\cdot}01$ compared with $0{\cdot}020 > P > 0{\cdot}01$). This result is directly analogous to the sex difference in mortality due to melanoma for the 3 corresponding states of Queensland, New South Wales and Victoria. Beardmore (1972) has demonstrated that when age-standardized death rates are calculated, the greater contribution by males diminishes the further the States are situated from the equator.

It is therefore plausible to suggest that, under appropriate conditions, sunlight may exert a carcinogenic effect on skull bones infiltrated by Paget's disease. Grady *et al.* (1943) reported a mean sarcoma:carcinoma induction ratio of 3:1 after UV irradiation of the ears of albino mice. Nearly monochromatic light of wavelength 254 nm is only weakly carcinogenic relative to longer wavelengths of up to 320 nm (Blum & Lippincott, 1942). Since the intensity of this spectrum of UV radiation is greater (Gates, 1966) and women are more prone to take protective measures for sunburn, at lower than at higher latitudes, a climatically dependent sex difference in sarcoma induction might be anticipated.

Bloomfield (1977) has commented on two aspects of the English data of Price & Goldie (1969), namely, a right-sided preponderance of Paget's sarcoma and the almost exclusive occurrence of pelvic disease in males. His small Tasmanian series showed the same trend. However, neither of these observations reached significant proportions in any individual or the combined national series.

Among the important variables associated with the age at presentation with sarcoma, the younger American cases (allowing for sex) and the younger female cases (allowing for country) are probably due to demographic characteristics, whereby the mean population age is lower in the U.S.A. and women exceed men in all 3 countries at ages at risk to contract Paget's disease. The more interesting and original result is the observation that, for the combined series, after due allowance for the effects of country and sex, patients with affected feet, legs and pelvis present at an average of 2·4 years later than those whose bones are affected elsewhere. Whether this finding is related to such factors as walking, weight-bearing or previous fracture remains to be elucidated.

### REFERENCES

BARRY, H. C. (1969) *Paget's Disease of Bone*. Edinburgh: Livingstone.

BEARDMORE, G. L. (1972) The epidemiology of malignant melanoma in Australia. In *Melanoma and Skin Cancer*. Ed. W. H. McCarthy. Sydney: Australian Cancer Society. p. 39.

BLOOMFIELD, J. A. (1977) Paget's sarcoma in Tasmania. *Med. J. Aust.*, **2**, 49.

BLUM, H. F. & LIPPINCOTT, S. W. (1942) Carcinogenic effectiveness of ultraviolet radiation of wavelength 2537A. *J. Natl Cancer Inst.*, **3**, 211.

BOYD, J. T., DOLL, R., HILL, G. B. & SISSONS, H. A. (1969) Mortality from primary tumours of bone in England and Wales, 1961–63. *Br. J. Prev. Soc. Med.*, **23**, 12.

COLEY, B. L. (1960) *Neoplasms of Bone and Related Conditions*. New York: Hoeber. 2nd edn. p. 753.

COLLINS, D. H. (1956) Paget's disease of bone incidence and subclinical forms. *Lancet*, ii, 51.

CONES, D. M. T. (1953) An unusual bone tumour complicating Paget's disease. *J. Bone Jt Surg.*, **35B**, 101.

DICKSON, D. D., CAMP, J. D. & GHORMLEY, R. K. (1945) Osteitis deformans: Paget's disease of the bone. *Radiology*, **44**, 449.

FEINERMAN, L. K. & SHAPIRO, L. (1970) Osteogenic sarcoma: report of a case arising in Paget's disease of the bone with extension to the scalp. *Int. J. Dermatol.*, **9**, 96.

GATES, D. M. (1966) Spectral distribution of solar radiation at the Earth's surface. *Science*, **151**, 523.

GRADY, H. G., BLUM, H. F. & KIRBY-SMITH, J. S. (1943) Types of tumor induced by ultraviolet radiation and factors influencing their relative incidence. *J. Natl Cancer Inst.*, **3**, 371.

GRININGER, D. R. & RIGLER, R. G. (1968) Lymphatic metastases from Paget's sarcoma. *Geriatrics*, **23**, 97.

JAFFE, H. L. (1933) Paget's disease of bone. *Arch. Pathol.*, **15**, 83.

LARSSON, S. E. & LORENTZON, R. (1974) The geographic variation of the incidence of malignant primary bone tumours in Sweden. *J. Bone Jt Surg.*, **56A**, 592.

MANEY, A. W. (1952) Lymphatic dissemination of a sarcoma superimposed on Paget's disease of the os calcis. *Br. J. Surg.*, **40**, 84.

MCKENNA, R. J., SCHWINN, C. P., SOONG, K. Y. & HIGINBOTHAM, N. L. (1964) Osteogenic sarcoma arising in Paget's disease. *Cancer*, **17**, 42.

PAGET, J. (1882) Additional cases of osteitis deformans. *Trans. R. Med.-Chir. Soc.* (*London*), **65**, 225.

PAGET, J. (1889) Remarks on osteitis deformans. *Illustr. Med. News.*, **2**, 181.

PIKE, M. M. (1943) Paget's disease with associated osteogenic sarcoma. *Arch. Surg.*, **46**, 750.

PRICE, C. H. G. (1962) The incidence of osteogenic sarcoma in south-west England and its relationship to Paget's disease of bone. *J. Bone Jt Surg.*, **44B**, 366.

PRICE, C. H. G. & GOLDIE, W. (1969) Paget's sarcoma of bone. A study of eighty cases from the Bristol and Leeds bone tumour registries. *J. Bone Jt Surg.*, **51B**, 205.

PRICE, C. H. G. & JEFFREE, G. M. (1977) Incidence of bone sarcoma in south-west England, 1946–74, in relation to age, sex, tumour site and histology. *Br. J. Cancer*, **36**, 511.

RODGER, R. C., THOMPSON, R. K. & WAGNER, J. A. (1951) Osteogenic sarcoma arising in Paget's disease of calvarium. *Bull. Sch. Med. Maryland*, **36**, 19.

ROSENKRANTZ, J. A., WOLF, J. & KAICHER, J. J. (1952) Paget's disease (osteitis deformans). Review of one hundred and eleven cases. *Ann. Intern. Med.*, **90**, 610.

SCHMORL, G. (1932) Uber Osteitis deformans Paget. *Virchows Arch. Pathol. Anat.*, **283**, 694.

SINGER, F. R. (1977) *Paget's Disease of Bone*. New York: Plenum Press.

VILLIAUMEY, J. & LARGET-PIET, B. (1974) Le dégénérescence sarcomateuse de l'os pagétique. In *La Maladie de Paget*. Ed. D. J. Hioco. International Symposium, Laboratiore Armour Montagu, Paris.

### APPENDIX: SOURCES OF MATERIAL

(Numbers of cases in parentheses)

*Australia*

BARRY, H. C. (1969) *Paget's Disease of Bone*. Edinburgh: Livingstone. (119)

BLOOMFIELD, J. A. (1977) *Med. J. Aust.*, ii, 49. (6)

LAKE, M. (1951) *J. Bone Jt Surg.*, **33B**, 323. (7)

SEAR, H. R. (1949) *Br. J. Radiol.*, **22**, 580. (5)

SYME, J. (1969) *Aust. Radiol.*, **13**, 219. (1)

*United Kingdom*

BRAILSFORD, J. F. (1938) *Br. J. Radiol.*, **11**, 507. (1)

CONES, D. M. T. (1953) *J. Bone Jt Surg.*, **35B**, 101. (1)

DAVIE, T. B. & COOKE, W. E. (1937) *Br. J. Surg.*, **25**, 299. (2)

FAIRBANK, H. A. T. (1950) *J. Bone Jt Surg.*, **32B**, 253. (1)
GALBRAITH, H. B., EVANS, E. C. & LACEY, J. (1977) *Postgrad. Med. J.*, **53**, 33. (1)
HARNETT, W. L. (1952) *A Survey of Cancer in London. Br. Emp. Cancer Campaign*, p. 676. (2)
MANEY, A. W. (1952) *Br. J. Surg.*, **40**, 84. (1)
MCKILLOP, J. H., FOGELMAN, I., BOYLE, I. T. & GREIG, W. R. (1977) *J. Nucl. Med.*, **18**, 1039. (1)
PLATT, H. (1947) *Br. J. Surg.*, **34**, 232. (8)
PRICE, C. H. G. & GOLDIE, W. (1969) *J. Bone Jt Surg.*, **51B**, 205. (80)
PYGOTT, F. (1957) *Lancet*, i, 1170. (3)
ROSS, F. G. M., MIDDLEMISS, J. H. & FITTON, J. M. (1973) *Bone—Certain Aspects of Neoplasia*. Eds. C. H. G. Price & F. G. M. Ross. London: Butterworth. p. 41. (1)
RUSSELL, D. S. (1949) *J. Bone Jt Surg.*, **31B**, 281. (3)
SEMPLE, J. C. (1969) *Postgrad. Med. J.*, **45**, 740. (1)
SHANNON, F. T. & HOPKINS, J. S. (1977) *Acta Orthop. Scand.*, **48**, 385. (1)
SISSONS, H. A. (1976) *Bones and Joints*. Eds. L. V. Ackerman & H. J. Spjut. Baltimore: Williams and Wilkins. p. 154. (1)
STEVENS, J. & LENNOX, B. (1958) *J. Bone Jt Surg.*, **40B**, 735. (1)
STRANGE, F. G. S. (1951) *Proc. R. Soc. Med.*, **44**, 252. (1)
STRICKLAND, B. (1959) *Br. J. Radiol.*, **32**, 705. (2)
TAPP, E. (1967) *Postgrad. Med. J.*, **43**, 436. (1)
WALTON, I. G. & STRONG, J. A. (1973) *Lancet*, i, 887. (1)

*United States*

ACKERMAN, L. V. & SPJUT, H. J. (1962) *Atlas of Tumor Pathology*. Washington: Armed Forces Institute of Pathology. p. 140. (1)
ADLER, H. & EICHNER, G. (1958) *Am. J. Roentgenol.*, **79**, 648. (1)
AGAR, D. F. & WARREN, S. (1948) *J. Missouri Med. Ass.*, **45**, 348. (1)
ALBEE, F. H. (1936) *J. Am. Med. Ass.*, **107**, 1693. (1)
ANDERSON, J. T. & DEHNER, L. P. (1976) *J Bone Jt Surg.*, **58A**, 994. (2)
BAILEY, R. W. & STEVENS, D. B. (1961) *J. Bone Jt Surg.*, **43A**, 845. (1)
BIRD, C. E. (1927) *Arch. Surg.*, **14**, 1187. (8)
BOUTOURAS, G. D. & GOODSITT, E. (1963) *J. Int. Coll. Surg.*, **40**, 380. (2)
BRESLICH, P. J. (1931) *Arch. Surg.*, **23**, 813. (1)
CAMP, J. D. (1925) *Radiology*, **5**, 495. (2)
CAMPBELL, E. & WHITFIELD, R. D. (1943) *N.Y. St. J. Med.*, **43**, 931. (3)
CARMAN, R. D. & CARRICK, W. M. (1921) *J. Radiol.*, **2**, 7. (1)
CASE RECORD (1926) *Boston Med. Surg. J.*, **95**, 1215. (1)
COLEY, B. L. & SHARP, G. S. (1931) *Arch. Surg.*, **23**, 918. (3)
COURVILLE, C. B., DEEB, P. & MARSH, C. (1962) *Bull. Los Angeles Neurol. Soc.*, **27**, 57. (3)
DERMAN, H., PIZZOLATO, P. & ZISKIND, J. (1951) *Am. J. Roentgenol.*, **65**, 221. (1)
DETENBECK, L. C., SIM, F. H. & JOHNSON, E. W. (1973) *J. Am. Med. Ass.*, **224**, 213. (8)
DICKSON, D. D., CAMP, J. D. & GHORMLEY, R. K. (1945) *Radiology*, **44**, 449. (3)
ESPOSITO, W. J. & BERNE, A. S. (1960) *Am. J. Roentgenol.*, **83**, 698. (2)
FINNESON, B. E., GOLUBOFF, B. & SHENKIN, H. A. (1958) *Neurology*, **8**, 82. (1)
FREYDINGER, J. E., DUHIG, J. T. & MCDONALD, L. W. (1963) *Arch. Path.*, **75**, 496. (7)
GOLDENBURG, R. R. (1954) *J. Med. Soc. N.J.*, **51**, 106. (1)
GRADER, J. & MOYNIHAN, J. W. (1961) *J. Am. Med. Ass.*, **176**, 685. (1)
GRININGER, D. R. & RIGLER, R. G. (1968) *Geriatrics*, **23**, 97. (2)
HANSEN, T. L. (1942) *Trans. West. Surg. Ass.*, **51**, 59. (1)
HUTTER, R. V. P., FOOTE, F. W., FRAZELL, E. L. & FRANCES, K. C. (1963) *Cancer*, **16**, 1044. (2)
JAFFE, H. L. (1958) *Tumors and Tumorous Conditions of the Bone and Joints*. Philadelphia: Lea and Febiger. p. 464. (5)
KARPAWICH, A. J. (1958) *Oral Surg.*, **11**, 827. (1)
KELLER, M. J. & CAMARDA (1964) *St. Vincent's Hosp. Med. Bull. (Bridgeport)*, **6**, 9. (1)
KINTNER, E. P. (1951) *J. Indiana Med. Ass.*, **44**, 304. (1)
KIRSCHBAUM, J. D. (1943) *Arch. Path.*, **36**, 74. (1)
KNIGHTS, E. M., JR (1951) *R.I. Med. J.*, **34**, 258. (1)
LICHTENSTEIN, L. (1975) *Diseases of Bone and Joints*, 2nd ed. St. Louis: Mosby. p. 188. (1)
LODWICK, G. S. (1971) *Atlas of Tumor Radiology*. Ed. P. J. Hodes. Chicago: Year Book Medical Publishers. p. 430. (1)
MANGANIELLO, L. O. J., REIMANN, D. L. & WAGNER, J. A. (1948) *Arch. Neurol. Psychiat. (Chic.)*, **59**, 99. (1)
MCKENNA, R. J., SCHWINN, C. P., SOONG, K. Y. & HIGINBOTHAM, N. L. (1964) *Cancer*, **17**, 42. (33)
MINER, I. E. (1950) *Bull. Hosp. Jt Dis. (N.Y.)*, **11**, 26. (6)
NEWMAN, F. W. (1946) *J. Bone Jt Surg.*, **28**, 798. (2)
NICHOLAS, J. A. & KILLORAN, P. (1965) *J. Bone Jt Surg.*, **47A**, 450. (5)
OCHSNER, A. & GAGE, I. M. (1930) *Surg. Clin. N. Am.*, **10**, 851. (1)
OJEMANN, R. G. & JAIN, K. K. (1963) *J. Neurosurg.*, **20**, 471. (2)
PANCOAST, H. K., PENDERGRASS, E. P. & SCHAEFFER, J. P. (1940) *The Head and Neck in Roentgen Diagnosis*. Springfield: Thomas. p. 243. (1)
PERLMAN, R. (1934) *J. Bone Jt Surg.*, **16**, 594. (1)
PIKE, M. M. (1943) *Arch. Surg.*, **46**, 750. (2)
PORRETTA, C. A., DAHLIN, D. C. & JANES, J. M. (1957) *J. Bone Jt Surg.*, **39A**, 1314. (16)
ROSENMERTZ, S. K. & SCHARE, H. J. (1969) *Oral Surg.*, **28**, 304. (1)
SADAR, E. S., WALTON, R. J. & GOSSMAN, H. H. (1972) *J. Neurosurg.*, **37**, 661. (1)
SCHATZKI, S. C. & DUDLEY, H. R. (1961) *Cancer*, **14**, 517. (20)
SCHWAMM, H. A. (1971) *Milit. Med.*, **136**, 895. (1)
STERN, W. E. (1969) *Bull. Los Angeles Neurol. Soc.*, **34**, 221. (1)
SUMMEY, T. J. & PRESSLY, C. L. (1946) *Ann. Surg.*, **123**, 135. (2)
THOMPSON, J. B., PATTERSON, R. H. JR. & PARSONS, H. (1970) *J. Neurosurg.*, **32**, 534. (7)
WARREN, S. (1948) *J. Missouri Med. Ass.*, **45**, 348. (1)
WOLFE, A. M. & BLACK, W. C. (1940) *Rocky Mt Med. J.*, **37**, 586. (1)
WOODARD, H. Q. (1959) *Cancer*, **12**, 1226. (6)
YOKOO, H., SUCKOW, E. E. & BROCK, D. R. (1961) *Quart. Bull. Northw. Univ. Med. Sch.*, **35**, 48. (1)